# Comparison of EndoPredict and Oncotype DX Test Results in Hormone Receptor Positive Invasive Breast Cancer

**DOI:** 10.1371/journal.pone.0058483

**Published:** 2013-03-07

**Authors:** Zsuzsanna Varga, Peter Sinn, Florian Fritzsche, Arthur von Hochstetter, Aurelia Noske, Peter Schraml, Christoph Tausch, Andreas Trojan, Holger Moch

**Affiliations:** 1 Institute of Surgical Pathology, University Hospital Zurich, Zurich, Switzerland; 2 Institute of Pathology, University Hospital Heidelberg, Heidelberg, Germany; 3 Pathology Institute Enge, Zurich, Switzerland; 4 Breast Cancer Center Seefeld, Zurich, Switzerland; University Medical Centre Utrecht, The Netherlands

## Abstract

**Aim:**

Several multigene expression-based tests offering prognostic and predictive information in hormone-receptor positive early breast cancer were established during the last years. These tests provide prognostic information on distant recurrences and can serve as an aid in therapy decisions. We analyzed the recently validated reverse-transcription-quantitative-real-time PCR-based multigene-expression Endopredict (EP)-test on 34 hormone-receptor positive breast-cancer cases and compared the EP scores with the Oncotype DX Recurrence-scores (RS) obtained from the same cancer samples.

**Methods:**

Formalin-fixed, paraffin-embedded invasive breast-cancer tissues from 34 patients were analyzed by the EP-test. Representative tumor blocks were analyzed with Oncotype DX prior to this study. Tumor tissue was removed from unstained slides, total-RNA was isolated and EP-analysis was performed blinded to Oncotype DX results.

**Results:**

Extraction of sufficient amounts of RNA and generation of valid EP-scores were possible for all 34 samples. EP classified 11 patients as low-risk and 23 patients as high-risk. RS Score defined 15 patients as low-risk, 10 patients as intermediate-risk in and 9 patients as high-risk. Major-discrepancy occurred in 6 of 34 cases (18%): Low-risk RS was classified as high-risk by EP in 6 cases. Combining the RS intermediate-risk and high-risk groups to a common group, the concordance between both tests was 76%. Correlation between continuous EP and RS-scores was moderate (Pearson-coefficient: 0.65 (p<0.01).

**Conclusion:**

We observed a significant but moderate concordance (76%) and moderate correlation (0.65) between RS and EP Score. Differences in results can be explained by different weighting of biological motives covered by the two tests. Further studies are needed to explore the clinical relevance of discrepant test results with respect of outcome.

## Introduction

Biomarkers in breast cancer contribute essentially to adjuvant and preoperative therapy assessment. Additionally to conventional prognostic factors as tumor-size, grading or nodal status, treatment decisions include the three established predictive biomarkers as estrogen (ER) and progesterone (PR) receptors and the HER2 status [1], [2], [3]. Prognostic factors provide information on the likelihood of cancer progression in untreated patients, whereas predictive factors carry information on the probability of therapy response [3], [4]. Multigene assays have become more widely used to prognosticate breast cancer clinical course and assist in the decision making for or against adjuvant chemotherapy [5], [6], [7], [8], [9], [10]. The benefit of chemotherapy in addition to regular hormonal therapy remains a subject of dispute in hormone receptor positive early breast cancer [3], [11], [12]. Several tests were developed in the recent years measuring the expression profile of cancer-related genes and providing prognostic information on disease-free and overall survival. The Netherlands Cancer Institute in Amsterdam launched Mammaprint, a 70-gene assay in 2002. The genetic signature of Mammaprint predicted metastasis free survival and overall survival in a validation study on 295 breast cancer patients [13], [14]. Oncotype DX, a 21-gene assay was first tested in clinical trials in 2004. It is able to quantify the likelihood of distant recurrence and the probability of response to chemotherapy in early breast cancer [11], [15], [16]. The Recurrence Score was validated in the NSABP B-14 trial in 2004 on 645 patients. The NSABP B-20 trial in 2006 analyzed 651 patients and validated the benefit of additional chemotherapy in patients with high Recurrence Score [15], [16]. Since the initiation of Mammaprint and Oncotype DX, additional multigene tests (e.g. Breast Cancer Index, Rotterdam, Invasiveness gene signature, PAM5) were developed, which are being either commercially available or currently under clinical investigation [17]. These gene assays, either reverse transcription-quantitative real-time PCR (RT-qPCR)- or microarray-based increasingly meet clinical attention, as they represent potential additional tools to conventional pathological prognostic factors and to established international oncological guidelines [17]. In 2011, a new 12-gene test, the EndoPredict assay was launched. It was validated independently in patients from two large randomized phase III trials (Austrian Breast and Colorectal Cancer Study Group (ABCSG)-6: n = 378, ABCSG-8: n = 1324) [18]. The EndoPredict (EP) risk score provided additional prognostic information to the risk of distant recurrence in hormone receptor positive, nodal negative breast cancer patients. The EPclin score which is the EP score combined with the clinico-pathological parameters tumor size and nodal status was the first RNA-based prognostic test for breast cancer to outperform all conventional clinic-pathologic risk factors alone or in combination with each other [18], [19]. The performance of the EndoPredict assay in decentralized testing using formalin-fixed, paraffin-embedded (FFPE) tumor tissue was successfully shown in seven European pathology institutions reaching 100% concordance between the different sites [19].

In this retrospective study, we addressed to investigate the concordance of EndoPredict scores and the Oncotype DX Recurrence Scores in 34 hormone receptor positive breast cancer patients.

## Materials and Methods

### Patients’ Characteristics

34 patients with invasive breast carcinoma were selected for this study (18 cases from the Institute of Surgical Pathology, University Hospital Zurich, Switzerland, 10 cases from the Institute of Pathology, University Hospital Heidelberg, Germany and 6 cases from the Pathology Institute Enge Zürich Switzerland). The time of diagnoses was between 2008–2012.

All tumors were estrogen receptor and in the majority also progesterone receptor positive. On histology 28 tumors corresponded to invasive ductal carcinoma (82%), three to invasive lobular carcinoma (9%) and three tumors were diagnosed as a mixed invasive carcinoma with ductal, lobular and squamous components (9%).

Clinico-pathological data of the tumors are summarized in **Table 1**.

**Table 1:** Summary of clinical data.

n = 34		
Age (years)	<40	3
	>40	31
Tumor size	pT1b (0.5 to 1 cm)	5
	pT1c (>1 to 2 cm)	19
	pT2 (>2 to 5 cm)	8
	pT3 (>5 cm)	2
Nodal status	negative	21
	positive	13
Grading	1	2
	2	21
	3	11
ER status	positive	34
	Negative	–
PR status	positive	31
	negative	3
HER2 status	negative	33
	positive	1

ER: estrogen receptors, PR: progesterone receptors, NA: not available.

The study was designated and approved as a quality control study by the Review Board of the Institute of Surgical Pathology (project Nr. 285). The review board specifically waived from the need of an approval of the cantonal ethical committee. According to the Federal Swiss Law for research and as required by the ethical committee of Canton Zurich, no additional ethical committee approval was necessary, as the study was designated as a quality control study and all tissue samples were analyzed in a completely anonymized way.

### Immunohistochemistry for ER/PR/HER2 and Ki-67

Hormone receptor status (in all cases), HER2 status (in 16 cases, from Heidelberg and Pathology Enge) and proliferation fraction (in 33 cases) were determined during routine histological diagnostics using commercial antibodies following the manufactures’ recommendations on the Ventana Benchmark and Leica Bond autostainers. Primary antibodies were detected using the iVIEW DAB detection kit and the signal was enhanced using the amplification kit. Following markers and dilutions were used: HER2 (4B5 Ventana Basel Switzerland) (MIB-1 (Ki-67) (DAKO Denmark, Glostrup, dilution 1∶20), estrogen receptors (6F11, Ventana Basel, Switzerland, dispenser), progesterone receptor (1A6, Ventana, Basel Switzerland, dispenser) as described previously [20]. Cut-off for positive ER/PR status was set as 1% of positively stained nuclei. HER2 immunohistochemstry was scored as described in the ASCO guidelines [21].

### Fluorescence in situ Hybridization (FISH) for HER2

HER2 status was determined within on the primary tumor using FISH only-methodology in 18 of 34 cases (cases from Zurich, University Hospital). All procedures for the FISH analyses were carried out by following the recommended protocol of the manufacturers using a dual fluorescence kit (PathVysion™, Vysis, Abbott AG, Diagnostic Division Baar, Switzerland).The reactions were evaluated using an Olympus computer guided fluorescence microscope (BX61, Olympus Schweiz AG, Volketswil, Switzerland). FISH testing was evaluated in reference to the ASCO guidelines [21].

Cut-off for positive *HER2* status was set as *HER2*/*CEP17* ratio ≥2.2.

### Tissue Preparation for Oncotype DX Tests

Upon request of the oncologists in charge, samples were submitted to Genomic Health (Redwood City, CA) for Oncotype DX testing for breast cancer prior to this study. For this assay, one representative paraffin block was chosen from the cases, containing the largest amount of invasive tumor cells on the hematoxyline & eosin (H&E) slides. The amount of tumor cells was at least 10% of the H&E slide. According to the pathology guidelines of Oncotype DX, 15 unstained serial slides of 4 micrometer thickness per tumor block were freshly cut from the paraffin blocks and submitted for the assay. Recurrence Score were assessed by Genomic Health in all patients. RNA-based ER, PR and *HER2* status were available in 33 of 34 patients.

### Tissue Preparation and RNA Isolation for EndoPredict Tests

The same paraffin blocks assessed by Oncotype DX were used for EndoPredict. Slides and sections for the EndoPredict assay for this study contained immediately adjacent tissues to those previously submitted to Genomic Health. The amount of invasive carcinoma tissue was at least 10% of the whole section surface in each case.

One H&E section and three adjacent serial unstained slides (4 µm) were cut from each paraffin block. On the H&E slide, the area of the invasive tumor cells was identified under light microscope and marked with ink. The same area was also marked on the unstained slides. Tumor tissue was scraped from the unstained slides into a plastic tube using a scalpel permitting the analysis of almost 100% of invasive tumor tissue by the EndoPredict test. Total RNA was extracted using a silica-coated magnetic bead-based method as previously described RNA was eluted with 100 µL elution buffer and subjected to DNase digestion as described to get DNA-free total RNA.

Unstained slides and H&E sections for both Oncotype DX and EndoPredict analysis were prepared in an identical way in the Institute of Surgical Pathology, University Hospital Zurich.

### Performance of EndoPredict Test

The EndoPredict assay (Sividon Diagnostics, Cologne, Germany) was performed as published previously [19]. In brief, expression of 8 genes–of-interest (*AZGP1, BIRC5, DHCR7, IL6ST, MGP, RBBP8, STC2, UBE2C*) and three reference genes (*CALM2, OAZ1, RPL37A*) as well as the amount of residual genomic DNA (*HBB*) were assessed by one-step RT-qPCR using the SuperScript III PLATINUM One-Step Quantitative RT-PCR System with ROX (Invitrogen, Karlsruhe, Germany) according to manufacturer’s instructions in a VERSANT® kPCR Molecular System (Siemens Healthcare Diagnostics). Sequences of primers and FAM/TAMRA-labeled probes were published previously [18]. EP and EPclin scores as well as classification into low or high risk of distant metastasis were calculated from analytical PCR results, tumor size and nodal status using a web-based implementation as described previously [19]. RT-qPCR analyses and calculations of EP and EPclin scores were performed by laboratory scientists in Sividon Diagnostics blinded to the results from the Oncotype DX tests. The scores and risk groups for each patient were subsequently transferred for analysis to one pathologist (Z.V). Extraction of a sufficient amount of RNA and generation of a valid EP score was possible for all 34 study samples.

### Statistics

Statistical analysis was performed using the Pearson’s correlation coefficient. Signifcance was defined as p<0.05.

## Results

### Oncotype DX Recurrence Score (RS)

Results of the individual patients were provided by Genomic Health to the submitting clinicians (A.T, C.T) and to the pathologists (A.N, Z.V, P.S, F.F, A.H).

Recurrence Score (RS) revealed low risk in 15 patients, intermediate risk in 10 patients and high risk in 9 patients.

### EndoPredict Test

The EndoPredict test results in an EP risk score and an EPclin score.

According to the **EP** risk score 11 patients were classified as low risk and 23 patients as high risk.

The EPclin score (combining EP risk score with tumor size and nodal status) re-classified 8 of the 23 EP high risk patients into the low risk group resulting in 19 patients with low and 15 patients with high risk of distant metastasis.

### Correlation and Concordance between Recurrence Score and EP Risk Score

Comparing EP risk scores with Recurrence Score, a moderate (yet significant) correlation was found as reflected by a Pearson coefficient of 0.65 (p<0.01). Nine of 15 of samples classified as low risk by the Recurrence Score were also low risk by EP score (60%). Nine of nine RS high risk samples were also EP high risk (100%). By combining the Oncotype DX intermediate risk and high risk groups to one high risk group, the concordance of classification in low or high risk between both tests was found in 26 of 34 cases (76%).

**Figure 1:**
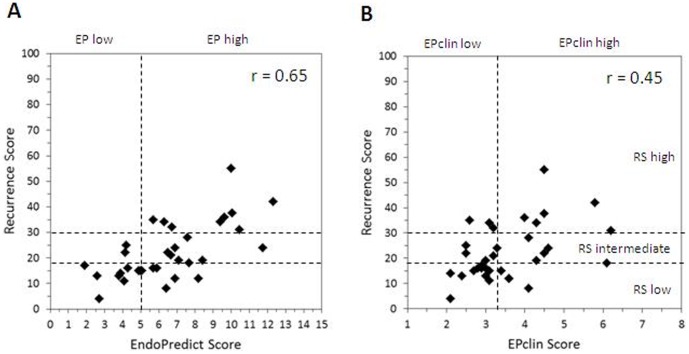
Analytical comparison of Recurrence Score with EndoPredict Score (A) and EPclin Score (B). r = Pearson coefficient.

The results of RS and EP scores are summarized in **Tables 2** **,** **3** **and** **Fig. 1A** **.**

**Table 2:** Comparison of EP score and Recurrence score (RS).

n = 34		Recurrence score (RS) (three tiered)		
		Low risk	Intermediate risk	High risk
EP score	Low risk	9 (26%)	2 (6%)	0 (0%)
	High risk	6 (18%)	8 (24%)	9 (26%)

RS in three tiered system.

**Table 3:** Comparison of EP score and Recurrence score (RS). RS in two tiered system: low vs. intermediate+high risk.

n = 34		Recurrence score (RS) (two tiered)	
		Low risk	High+Intermediate risk
EP score	Low risk	9 (26%)	2 (6%)
	High risk	6 (18%)	17 (50%)

### Correlation and Concordance between Recurrence Score and EPclin Score

Comparing the combined molecular-clinicopathologic EPclin score with the Recurrence Score the correlation was substantially smaller in comparison with the correlation between RS and EP scores. Pearson coefficient was 0.45 (p = 0.01).

Eleven of 15 samples classified as low risk by the Recurrence Score were also low risk by EPclin score (73%). Six of nine RS high risk samples were EPclin high risk (66%). Combining the Oncotype DX intermediate risk and high risk groups to one high risk group, the concordance of classification in low or high risk between both tests was detected in 22 of 34 cases (65%). The results of RS and EPclin score are summarized in **Tables 4** **,** **5** **and** **Fig. 1B** **.**

**Table 4:** Comparison of EPclin score and Recurrence score (RS). RS in three tiered system.

n = 34		Recurrence score (RS) (three tired)		
		Low risk	Intermediate risk	High risk
EPclin score	Low risk	11 (32%)	5 (15%)	3 (9%)
	High risk	4 (11%)	5 (15%)	6 (18%)

**Table 5:** Comparison of EPclin score and Recurrence score (RS). RS in two tiered system: low vs. intermediate+high risk.

n = 34		Recurrence score (RS) (two tiered)	
		Low risk	High+Intermediate risk
EPclin score	Low risk	11 (32%)	8 (24%)
	High risk	4 (12%)	11 (32%)

### Correlation and Concordance of Ki-67 to EP Score, EPclin Score and RS

We could find a statistically significant but moderate correlation between the two molecular scores and proliferation index. No significant correlation was observed between the EPclin score and Ki-67. (Pearson coefficient varied as follows: to EP: 0.55 (p<0.0001), to EPclin: 0.24 (p = 0.16), to RS: 0.56 (p<0.0001).

**Figure 2:**
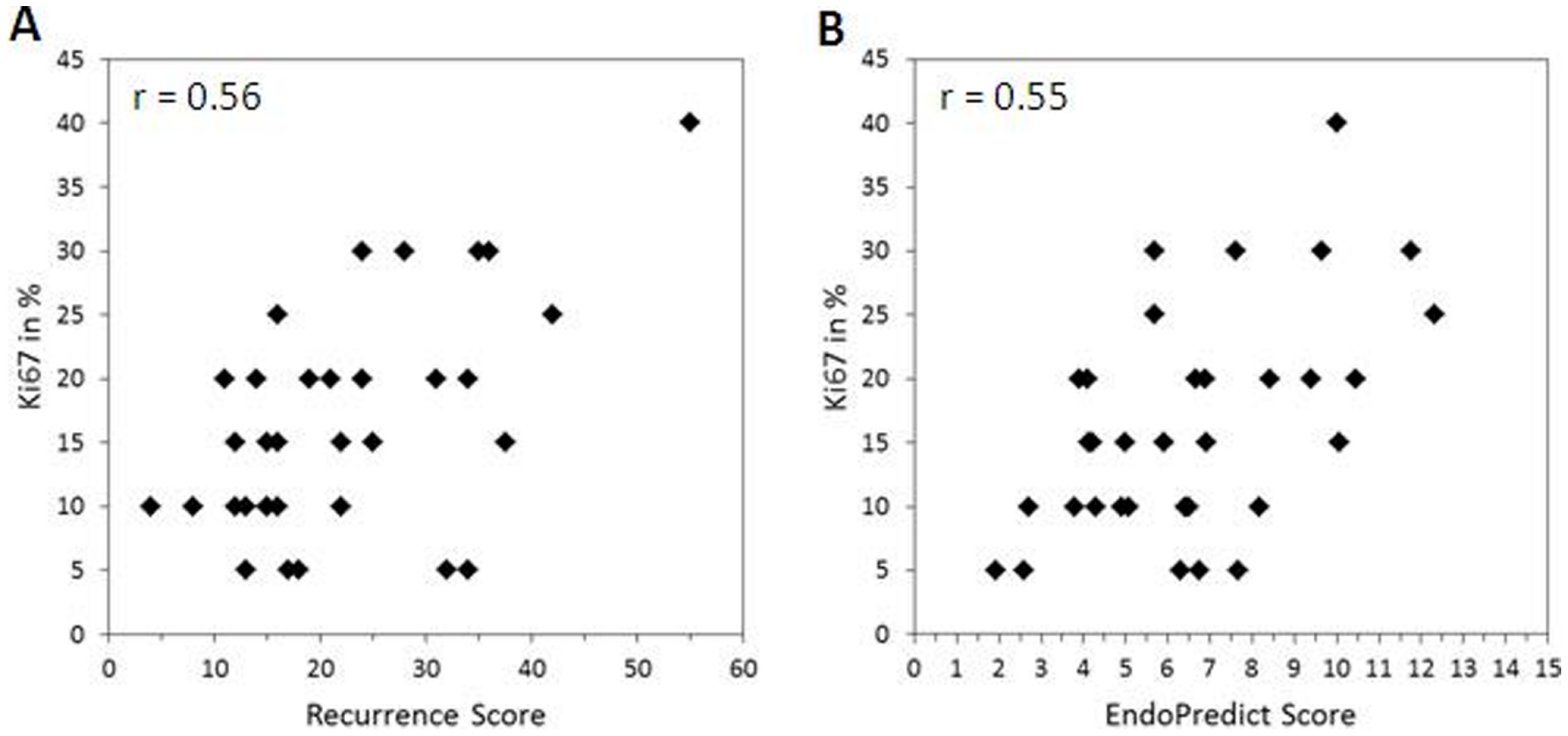
Analytical comparison of continuous Ki67 values with Recurrence Score (A) and EndoPredict Score (B). r = Pearson coefficient.

Results of continuous Ki-67 values and risk classes are illustrated in **Fig. 2**.

### Comparison of ER/PR/HER2 Status with Conventional Morphology and Oncotype DX Assay

We detected a *high concordance* in hormone receptor and HER2 status between conventional morphology and Oncotype DX testing.

33 of 33 patients were positive for ER with immunohistochemical (IHC) analysis and with Oncotype DX assay (100%).

28 of 33 patients had identical PR status with both methodologies (85%). Three patients had PR positive cells in approximately 20%–30% of the tumor cells on immunohistochemistry, which were assessed as negative with Oncotype DX. The re-analysis of the immunohistochemical PR reactions confirmed small amount of positively stained nuclei.

31 of 33 patients had matching HER2 status with both methods (94%). One patient had a negative HER2 status by FISH which was assessed as equivocal with Oncotype DX. Another patient had *HER2* amplification by FISH, which was negative by Oncotype DX. The FISH *HER2* reaction was re-analyzed again and the amplification status could be confirmed.

Results of hormone receptor/HER2 status and RS are summarized in **Table 6**.

**Table 6:** Comparison of ER/PR/*HER2* status with Oncotype DX assay and conventional methodology (Immunohistochemistry and fluorescence in situ hybridization ‘FISH’ testing).

n = 33			Oncotype DX testing		
Conventional metholodogy			positive	negative	equivocal
ER	positive	33 (100%)	33 (100%)	–	–
	negative	–	–	–	–
PR	positive	30 (91%)	26 (79%)	4 (12%)	–
	negative	3 (9%)	1 (3%)	2 (6%)	–
HER2	positive	1 (3%)	0	1 (3%)	–
	negative	32 (97%)	–	31 (94%)	1 (3%)
	equivocal	–	–	–	–

In one case results of Oncotype DX for ER/PR/HER2 status were not available.

## Discussion

In this study, we investigated retrospectively the correlation between EndoPredict scores and Oncotype DX Recurrence Scores using 34 hormone receptor positive breast cancer samples. Importantly, EndoPredict score showed a significant but only moderate correlation with the Recurrence Scores obtained by Oncotype DX testing. We found a moderate concordance of results regarding classification into risk groups between the two assays (reaching 76%) (two tiered). A major discrepancy between the two gene signatures was detected in 6 of 34 patients (18%) (three tiered).

The discrepancy and moderate correlation of the two molecular scores EP and RS might be due to differences in weighting of main biological motives covered by the genes included in the test algorithms such as proliferation or ER signaling. Some differences might be explained by the coverage of other motives, e.g. cell adhesion, invasion, or DNA repair [11], [18]. Interestingly, an even smaller agreement is achieved, if the molecular RS is compared to the combined molecular-clinico-pathological EPclin score as opposed to the molecular EP score. This lower agreement is likely caused by the fact that the EPclin score considers additional prognostic information that may not be reflected by the tumor’s RNA expression. Following the EPclin-based classification into low or high risk of metastasis would spare 19 of 34 (56%) patients a cytotoxic chemotherapy in the light of an estimated 10-years distant metastasis-free survival of 96% of EPclin low risk patients in the two clinical validation studies [18]. Nevertheless, further prospective clinical trials are needed to validate these results.

Another multi-gene test, Mammaprint, was previously been compared with Oncotype DX [22]. In this analysis, a higher concordance (81%) between high and intermediate risk groups from the Oncotype DX and poor prognostic groups of Mammaprint tests were shown. Our study showed a weaker concordance of 76% between EP sore and high/intermediate risk groups assessed by Oncotype DX.

Together, different multigene may result in different treatment recommendation for individual patients. One limitation of previous studies is the sample size. Further analyses with longer patient survival data are necessary for the re-validation of these results.

Oncotype DX is a RT-qPCR based 21-gene assay using *RNA* from FFPE tissue, comprising 16 cancer genes primarily related to tumor proliferation [11], [16]. This test is performed in a central reference laboratory. The NSABP B14 clinical trial validated, that patients with low RS developed significantly lower distant metastases than those patients with high RS [11]. Analysis on prognostic value of RS as to distant metastases in early hormone receptor positive breast cancer has been the subject of several further clinical studies since the NSABP B14 trial chemotherapy [6], [7], [8], [9], [10], [11], [12], [17].

The consecutive clinical trial, the NSABP B20 validated the predictive value of RS on additional chemotherapy on hormone receptor positive nodal negative breast cancer patients [15]. Data on 651 enrolled patients revealed that patients with high RS exhibited improved response to chemotherapy [10], [15]. On the other hand, it was also shown that patients with low risk RS did not benefit from additional chemotherapy [10], [15]. Response on chemotherapy in intermediate risk RS is currently being investigated in the ongoing TAILORx clinical trial [10].

The EndoPredict assay is an RT-qPCR-based 12-gene test using RNA from FFPE tissue, specifically validated in two clinical studies for recurrence prediction in hormone receptor positive, HER2 negative, nodal negative and positive breast cancer treated with adjuvant hormonal therapy alone [18]. The EP score provided significant prognostic information in addition to conventional prognostic clinico-pathological parameters such as tumor size, nodal status, grading, quantitative ER and Ki-67 as well as Adjuvant!Online [18]. Moreover, the combination of the molecular EP score with tumor size and nodal status to the comprehensive molecular-clinico-pathological EPclin score outperformed the established prognostic parameters in these two patients cohort [18]. Recently, it could be shown in a proficiency testing program including seven different pathological institutes that EndoPredict can be reliably performed in a decentralized setting in molecular pathological laboratories without the requirement of a reference lab [19].

In contrast to EndoPredict, Oncotype DX assay includes an RT-qPCR based determination of hormone receptor and HER2 gene amplification. This fact prompted several previous studies to compare expression profile of these parameters. Excellent correlation with 100% concordance has been reported by O’Connor et al. in a series on 80 breast cancer samples [23]. We found high concordance in ER/PR/HER2 status between Oncotype DX assay and established FISH or IHC assays, also regarded as a “gold standard”. There were only two discrepant cases for the HER2 status and six cases for progesterone receptors with no discrepant cases for estrogen receptors. This observation is in line with occasional false negative HER2 results reported as part of the Recurrence Score [24]. Importantly, Geradts et al. detected discrepancies in hormone receptor and HER2 status determined by the conventional assays (IHC and/or FISH) and RT-PCR methodologies. There was only 56 to 66% categorical concordance [25]. A similar result was found in a further study showing 33% of HER2 IHC-positive samples to be HER2 negative in RT-qPCR whereas the concordance of both methods in HER2 IHC-negative samples was 95% [26]. It is not clear at this time, which methodology is superior in respect of predictive power. This needs to be addressed in future prospective trials. In current clinical practice, such discrepancies in the most important predictive breast cancer biomarkers are significantly hampering the treatment decision making process. Interestingly, a strong correlation between morphological parameter (especially histological tumor grading) and Recurrences Score was established in a few previous studies [27], [28]. The classification into two-tiered (low and high) risk categories with EndoPredict assay can possibly yield in clearer separation of intermediate risk patients.

Concordance between Recurrence score and other prognostic assays or clinico-pathological parameter is of interest in clinical decision making.

Significant linear correlation between proliferation index (Ki-67) and Recurrence Score was established previously in hormone receptor positive breast cancer. These data recommend the potential use of more cost effective immunohistochemical assessment of proliferation fraction rather than ordering highly expensive Oncotype DX testing [29], [30]. Another study by Tang et al. found good independent prognostic information in tamoxifen treated patients when Recurrence Score and individual clinico-pathological parameter were analyzed together [31]. Interestingly, combining Adjuvant! Online recommendation with Recurrence Score did not provide better prognostic benefit in their analysis [31]. Recently, a good agreement of prognostic risk assignment between the gene expression-based “intrinsic” subtype test PAM50 and Oncotype DX was described [32].

Determining predictive markers with routine pathology assessment and using standardized reproducible criteria for morphological parameter (as grading, tumor size) represent a much less expensive alternative to multigene expression assays [10], [22]. It has been suggested that routine pathology markers are probably as reliable as genetic signatures at the current time, especially if combined mathematically [10], [22], [33]. We could detect significant but moderate correlation between continuous proliferation index (Ki-67) and RS and EP scores. This is at least partially due to the lack of standardization in assessing the Ki-67 index in breast cancer [34].

A considerable percentage of women diagnosed with breast cancer are aware of the valuable information multigene tests may add to their immediate therapeutic options [35], [36], [37]. The impact of Oncotype DX testing is clearly reflected on altered recommendations or therapy decision in view of RS, which reportedly varies from 19 to 44% of the studied patients [38], [39], [40], [41].

In conclusion, our data show moderate concordance between EndoPredict Score and Oncotyope DX results on individual patients. In the light of previous clinical and analytical validation data EP bears the promise to be an additional tool for decentralized multigene testing by local pathology with the advantage of the inclusion of important clinic-pathological data as nodal status. Further clinical studies are needed to compare both tests with regard to prediction of early and late distant metastasis, chemotherapy benefit and clinical outcome.
