# Correction: Comparison of EndoPredict and Oncotype DX Test Results in Hormone Receptor Positive Invasive Breast Cancer

**DOI:** 10.1371/annotation/f715f38e-7aee-4d2b-8bbf-da0411dc6ef3

**Published:** 2013-10-17

**Authors:** Zsuzsanna Varga, Peter Sinn, Florian Fritzsche, Arthur von Hochstetter, Aurelia Noske, Peter Schraml, Christoph Tausch, Andreas Trojan, Holger Moch

There was an error in the first sentence of the Funding Statement. The first sentence of the Funding Statement should read: "The funders of Sividion Diagnostics, Cologne, Germany conducted the Endopredict assay on all 34 tissue samples in a blinded way upon request of the authors." 

